# Diabetic Ketoacidosis Secondary to New Onset Type 1 Diabetes Mellitus Related to Pembrolizumab Therapy

**DOI:** 10.7759/cureus.13302

**Published:** 2021-02-12

**Authors:** Andrea Hernandez, Bassem Zeidan, Parth Desai, Johnathan Frunzi

**Affiliations:** 1 Internal Medicine, Medical Center of Trinity, Trinity, USA; 2 Internal Medicine, Medical Center of Trinity, Trinity , USA; 3 Critical Care Medicine, Medical Center of Trinity, Trinity , USA

**Keywords:** pembrolizumab, diabetic ketoacidosis, type i diabetes mellitus, pd1 receptor

## Abstract

Pembrolizumab is an immunoglobulin G4 (IgG4) monoclonal antibody used in the treatment of various types of cancers. Despite its efficacy, pembrolizumab does not specifically target cancer cells which often leads to common side effects seen in immunotherapies such as diarrhea, rash, fatigue, nausea, decreased appetite, pruritus, and endocrinopathies. Type 1 diabetes mellitus (T1DM) has been reported in 0.1% of the patients in pembrolizumab clinical trials. In this case report, we discuss a 65-year-old Caucasian male with a history of metastatic head and neck cancer that was previously treated with pembrolizumab and was subsequently admitted to the intensive care unit (ICU) due to new onset diabetic ketoacidosis (DKA). Based on the timing of his presentation and the pre-hospital/inpatient workup, notably a normal hemoglobin A1C (HbA1c) 72 hours prior to admission and a significant increase thereafter, it was concluded that his presentation of diabetic ketoacidosis was secondary to his most recent infusion of pembrolizumab. With immunotherapies like programmed cell death (PD1) receptor antibodies becoming a more common first-line treatment for various cancers, this case hopes to raise awareness about the possible endocrinologic-related adverse events to its use and may help guide outpatient management.

## Introduction

Pembrolizumab is an immunoglobulin G4 (IgG4) monoclonal antibody that binds to programmed cell death (PD1) receptors on T-cells and causes the immune system to kill tumor cells [1]. It has been found to be an effective adjuvant treatment of advanced cases of cancer such as metastatic melanoma, head and neck cancers, non-small cell lung carcinoma, gastric cancer, hepatocellular carcinoma, and renal cell carcinoma. The drug was the first antibody against PD1 to be approved by the Food and Drug Administration (FDA) for the treatment of metastatic or unresectable carcinomas [2]. Despite its efficacy, pembrolizumab does not specifically target cancer cells and may affect noncancerous cells, leading to common side effects seen in immunotherapies such as diarrhea, rash, fatigue, nausea, decreased appetite, and pruritus. There are other immune-related endocrine toxicities related to the use of PD1 antibodies, such as thyroid dysfunctions, adrenal insufficiencies, and hypophysitis, which are rare [3]. Less frequently, type 1 diabetes mellitus (T1DM) has been reported in 0.1% of the patients in clinical trials treated with pembrolizumab [4]. T1DM is commonly is diagnosed in children and young adults and rarely seen in adults over 40 years old. We will be discussing a patient who was previously treated with pembrolizumab and was admitted to the intensive care unit (ICU) due to new onset diabetic ketoacidosis (DKA).

## Case presentation

Our patient was a 67-year-old Caucasian male who presented to the emergency room with complaints of polydipsia, polyuria, lightheadedness, and generalized weakness. He had a past medical history of oligometastatic squamous cell carcinoma of the tongue to the right lung, generalized arthritis, deep vein thrombosis, essential hypertension, and right bundle branch block. Although he denied any history of diabetes, a review of his outpatient records revealed a recent diagnosis of pre-diabetes mellitus with a hemoglobin A1C (HbA1C) of 6.0% that was diagnosed three weeks after receiving his first infusion of pembrolizumab, in which he was supposed to begin taking metformin 500 mg two times a day.

On arrival, his vital signs were within normal limits except for a heart rate of 110 beats per minute (bpm). Examination of his oropharynx showed evidence of a previous dissection over the left lateral aspect of the tongue, causing his speech to be disarticulated but comprehendible. Poor skin turgor was also noted indicating dehydration. The rest of the physical examination was unremarkable. Initial laboratory findings were significant for hyperglycemia with a glucose level of 923 mg/dL and urinalysis significant for glucosuria and ketones. He was found to have a sodium of 132 mmol/L with a corrected sodium of 148 mmol/L, potassium of 7.3 mmol/L, chloride of 97 mmol/L, bicarbonate of 9 mmol/L, and an elevated anion gap of 26 mmol/L, which was consistent with diabetic ketoacidosis. He was also found to have an acute kidney injury with a blood urea nitrogen (BUN) of 75 mmol, and a creatinine of 3.0 mg/dL, likely due to severe dehydration, and also hypercalcemic with calcium of 10.8 mmol/L. More notably, a repeat HbA1C revealed 6.9% on this admission.

Consequently, the patient was admitted directly to the ICU and underwent aggressive treatment for severe diabetic ketoacidosis and dehydration. Insulin drip was started with half normal saline and blood glucose levels were measured hourly. His hyperkalemia was treated with calcium gluconate and kayexalate in addition to the insulin drip. After three days, the anion gap was closed and insulin drip was discontinued. He was then started on long-acting subcutaneous insulin. Five days later glucose levels had stabilized and he was then downgraded to the medical floor from the ICU. One week after his initial presentation, the patient was discharged from the hospital. 

## Discussion

While the incidence of T1DM was low in pembrolizumab clinical trials, the consequential development of T1DM is becoming increasingly recognized in the literature after recent treatments with pembrolizumab. We identified numerous isolated reports of checkpoint inhibitor-induced diabetes mellitus that can be found in the literature with pembrolizumab being implicated in six of those cases^ ^[4]. What makes this case distinct from previous case reports is that the other patients had received multiple infusions of pembrolizumab prior to onset of T1DM and symptomatic DKA. Our patient had received only one infusion three weeks prior which highlights this medication’s ability to react within the body. Furthermore, many of the cases we had reviewed did not have an HbA1C prior to immunotherapy infusion. While our patient’s HbA1C had been measured after his first pembrolizumab infusion, it was within normal limits only 48-72 hours prior to his presentation of DKA and a repeat HbA1C on admission showed a significant increase within such a short amount of time.

Our patient had metastatic squamous cell carcinoma of the head and neck with metastasis to the right lung that was initially diagnosed seven years prior to his presentation. His cancer was refractory to multiple interventions including chemotherapy, radiation, and surgeries. Despite these previous aggressive treatments, he tolerated them well without experiencing any life-threatening side-effects. The patient reported that his health rapidly deteriorated after he was started on pembrolizumab and stereotactic radiation therapy. Similar to the cases reported by Cheema et al. [4], our patient developed signs and symptoms consistent with DKA which was thought to be secondary to pembrolizumab-induced T1DM. Further investigation into his medical history was unrevealing of any other drug-related adverse events or any positive family history of diabetes. According to Clotman et al., the time to diagnose T1DM after initiation of anti-PD1 therapy averaged between one week to one year; the median time for development of T1DM was three infusions and a median age of 63 [5]. Our patient, at the age of 67, developed DKA three weeks after receiving his first infusion and experienced a rapid increase in his HbA1C to 6.9% on admission.

The exact mechanism of how pembrolizumab can lead to diabetes is unclear. Beta islet cells in the pancreas are responsible for the production of insulin. As beta islet cells contain programmed cell death ligand receptors (PD-L1), it is hypothesized that pembrolizumab binds to the T-cell’s LD1 receptors, inhibiting T-cell from binding to the PD-L1 on the beta islet cell and causes destruction of the beta islet cells as it is unable to recognize it as a non-cancerous cell [3]. This autoimmune response against pancreatic islet cells results in fulminant destruction over the short course of a few weeks. There is no special treatment for pembrolizumab-induced diabetes and ketoacidosis. Insulin therapy is the management of choice with no complications in the majority of cases. Similar to our case, it was noted that some patients encountered unpredictable hyperglycemic control and/or ketoacidosis episodes and required admission to the ICU [6].

## Conclusions

Although immunotherapies utilizing PD1 antibodies such as pembrolizumab have revolutionized the management of various cancers, this case demonstrates the growing concerns of developing major adverse events including endocrinopathy related side-effects. Prior to treatment with pembrolizumab, it is important to recommend routine check and monitoring of HbA1C, blood glucose and make patients aware of signs of diabetes and DKA. If identified early, this drug-related adverse effect can be managed appropriately and safely.
